# Networks of face-to-face social contacts in Niakhar, Senegal

**DOI:** 10.1371/journal.pone.0220443

**Published:** 2019-08-06

**Authors:** Gail E. Potter, Jimmy Wong, Jonathan Sugimoto, Aldiouma Diallo, John C. Victor, Kathleen Neuzil, M. Elizabeth Halloran

**Affiliations:** 1 The Emmes Company, Rockville, MD, United States of America; 2 California Polytechnic State University, San Luis Obispo, CA, United States of America; 3 Fred Hutchinson Cancer Research Center, Seattle, WA, United States of America; 4 Institut de Recherche pour le Développement, Niakhar, Senegal; 5 PATH, Seattle, WA, United States of America; 6 University of Maryland Center for Vaccine Development, Baltimore, MD, United States of America; 7 Department of Biostatistics, University of Washington, Seattle, WA, United States of America; Chinese University of Hong Kong, HONG KONG

## Abstract

We present the first analysis of face-to-face contact network data from Niakhar, Senegal. Participants in a cluster-randomized influenza vaccine trial were interviewed about their contact patterns when they reported symptoms during their weekly household surveillance visit. We employ a negative binomial model to estimate effects of covariates on contact degree. We estimate the mean contact degree for asymptomatic Niakhar residents to be 16.5 (95% C.I. 14.3, 18.7) in the morning and 14.8 in the afternoon (95% C.I. 12.7, 16.9). We estimate that symptomatic people make 10% fewer contacts than asymptomatic people (95% C.I. 5%, 16%; p = 0.006), and those aged 0-5 make 33% fewer contacts than adults (95% C.I. 29%, 37%; p < 0.001). By explicitly modelling the partial rounding pattern observed in our data, we make inference for both the underlying (true) distribution of contacts as well as for the reported distribution. We created an estimator for homophily by compound (household) membership and estimate that 48% of contacts by symptomatic people are made to their own compound members in the morning (95% CI, 45%, 52%) and 60% in the afternoon/evening (95% CI, 56%, 64%). We did not find a significant effect of symptom status on compound homophily. We compare our findings to those from other countries and make design recommendations for future surveys.

## 1 Introduction

Social contacts occurring in close proximity are transmission pathways for respiratory infections such as influenza [1]. These contacts form social networks over which the disease may spread, and estimation of network structures can help improve estimates of transmission parameters, predict disease spread, and refine social distancing strategies. Large-scale simulation models incorporating location-based social mixing patterns have been used to compare effectiveness of containment strategies for emerging pandemics [2–11]. Smaller-scale studies have incorporated more detailed network structure from social contact data collected in schools to assess the relevance of network structures to the spread of H1N1 pandemic in rural Pennsylvania [12] and to compare containment and and mitigation strategies for a pandemic [13, 14]. Effort is ongoing to collect social network data in multiple countries, identify the key network structures driving transmission, and incorporate these structures efficiently into simulation models as well as estimation methods. The relative roles of fomite transmission, large droplet transmission, and small droplet transmission are not fully understood; we focus in this paper on networks characterized by face-to-face interactions, which form pathways for large droplet transmission networks [15]. Analogous networks may be modelled for the other transmission routes.

One such network structure is homophily, the tendency to associate with others with similar characteristics [16]. The POLYMOD study measured network properties of eight European countries [17] and produced mixing matrices capturing age-based homophily as well as estimates of cross-generational contact patterns. These mixing matrices have been used to parametrize epidemiologic models of pertussis [18], to model the spread of norovirus gastroenteritis in Berlin [19], to estimate the impact of school closure on the transmission of close contact infections [20], and to predict spread of the H1N1 pandemic and compare effectiveness of vaccination strategies [21]. Furthermore, detailed census and demographic data have been used to create synthetic mixing matrices for 26 European countries to discuss how these social factors relate to epidemic patterns in the different countries [22]. In the POLYMOD study, a “contact” was defined as a two-way conversation of at least three words in the same location and/or a physical contact (such as a kiss or handshake). The survey analyzed in this paper defined “contact” as speaking with a person in the same location. If two people had conversations in three different locations, then three contacts were recorded.

Another key network property is the degree distribution, where the degree of a person is the number of contacts a person makes. Researchers have used analytic methods as well as simulation studies to demonstrate that degree distribution is an important factor in predicting the probability of an epidemic as well as the epidemic curve [23–25]. Measuring heterogeneity in degree helps assess the impact of “super-spreaders”, individuals who make large numbers of contacts and are important both for transmission and for targeted intervention strategies [26–28].

There has recently been a large effort to measure face-to-face social contact data in a variety of cultures and contexts. Such data may be collected by paper diaries, surveys, interviews, and/or electronic sensor data. The diary approach has been applied in multiple European countries [17, 29], Zimbabwe [30], Kenya [31], South Africa [32], Vietnam [33], Taiwan [34], and China [35]. Other studies have used surveys or interviews to collect data in the Netherlands and Thailand [36], Peru [37], Australia [38], and the United Kingdom [39]. Finally, electronic sensor data measuring close interactions has been collected in schools, workplaces, and in households [40–45] There are also efforts to collect combinations of survey data and electronic sensor data to obtain a more complete picture and to compare the different collection methods, on both small and large scales [46–49].

This study presents results from a social contact survey that was administered by in-person interviews in conjunction with a cluster-randomized influenza vaccine trial in Niakhar, Senegal. This is the first paper, to our knowledge, analyzing data from face-to-face contact networks in Senegal. We estimate key properties of the social network, including the degree distribution, homophily based on compound membership, and the location distribution of contacts. We compare our estimated network structures to those from networks analyzed in other countries and make recommendations for survey design.

## 2 Data

The Institut de Recherche pour le Développement (IRD) administered a brief contact survey in conjunction with an influenza vaccine trial conducted from 2009-2010 in Niakhar, Senegal, a region including 30 villages with approximately 40,000 residents at the start of the study. In this cluster-randomized trial, ten villages were assigned to receive an influenza vaccine campaign for children age 6 months to 10 years. Another ten villages received a similar vaccination campaign for children, but received inactivated poliovirus vaccine as a control vaccine. The remaining ten villages in the area did not receive a treatment program. In the twenty treated villages, the study staff made weekly surveillance visits to compounds. (A compound is a residence for an extended family, which may include multiple buildings housing different nuclear families; compounds form key locations for dense social interaction.) During these visits, all consenting compound members were asked whether they had experienced any influenza-associated symptoms (feverish, cough, sore throat, nasal congestion, or rhinorrhea) in the previous seven days and asked the onset date of symptoms. Those who reported symptoms received an influenza test and also responded to a brief social contact survey about their travel patterns and social contacts in the previous three days. Patients presenting to three health posts in the Niakhar region with influenza symptoms were surveyed as well. As such, this passive surveillance covered the entire region of 30 villages. This study, ClinicalTrials.gov NCT00893906, is closed, and the primary results for the trial have been published [50].

The form used to conduct the social contact survey is included in the supporting information (S1 Appendix). In this survey, respondents reported the number of people they contacted in their own compound on both the morning and the evening of the survey day. Next, they were asked whether they had visited a list of twelve geographic locations on the survey day, including up to five (non-home) compounds, a field, market, mosque/church, and others. For each location that was visited, respondents reported the time of day (AM, PM, or both) and the number of people contacted in that location. The same information was collected for the preceding two days. For children too young to respond to the survey, the questions were answered by a parent or guardian. This was left to interviewer discretion rather than defining an age cutoff. For this analysis, the number of contacts a participant made during a given time interval was calculated by summing reported numbers of contacts across locations that were visited.

Contact data were collected only from participants who reported they had experienced symptoms in the past seven days. However, respondents whose symptoms began on the survey date reported contact patterns for the previous two days, so provide some information about contact behavior while people are asymptomatic. Those whose symptoms began the day before the interview date gave contact information from one asymptomatic day and two symptomatic days. The incubation period of influenza is 1–4 days [51], so asymptomatic respondents may have been infectious.

Respondents were identified by their demographic surveillance system identification number. This number links all residents in the Niakhar region (including those who never reported symptoms) to a demographic database compiled by the IRD via quarterly censuses. The demographic information includes sex, age, ethnicity, and unique compound identification number, which allows calculation of compound size for each participant.

Ethics approval was obtained from the National Ethics Committee for Health Research (Senegal Ministry of Health and Social Welfare) and Western Institutional Review Board. The study was conducted in accordance with the principles of the Declaration of Helsinki (2008) and in compliance with Good Clinical Practice guidelines. Written consent was obtained for collection of the symptom and contact data.

## 3 Methods

In this section, we describe methods to characterize the following properties of the face-to-face social contact network:

1. The degree distribution, where the degree of a person is the number of contacts he/she makes (Section 3.1)
2. Homophily in compound membership (Section 3.3)
3. The location distribution of contacts (Section 3.4)

We discuss the impact of missing data on our estimates and describe the multiple imputation method we use to reduce bias in Section 3.2.

We restrict our analysis to surveys collected over the course of six months: August 1, 2009 to February 1, 2010, assuming the contact process is fairly constant over this time. During this time period, contact surveys were submitted for 77% of symptom reports. For participants who were ill multiple times during this period (and so submitted multiple surveys), we analyze the first submitted contact survey to reduce dependency in the data. A total of 3,758 surveys were included in our analysis.

Contacts that occurred on the day of the survey are subject to truncation bias, since the interview occurred before the day was over. Either AM or PM was recorded for interview time, but the exact time was not recorded, making it difficult to adjust for this truncation bias, particularly because the majority (97%) of interviews occurred in the morning. For this reason, we focus our analysis on contacts reported during the two days prior to the survey date.

### 3.1 Modelling the degree distribution

**Fig 1:**
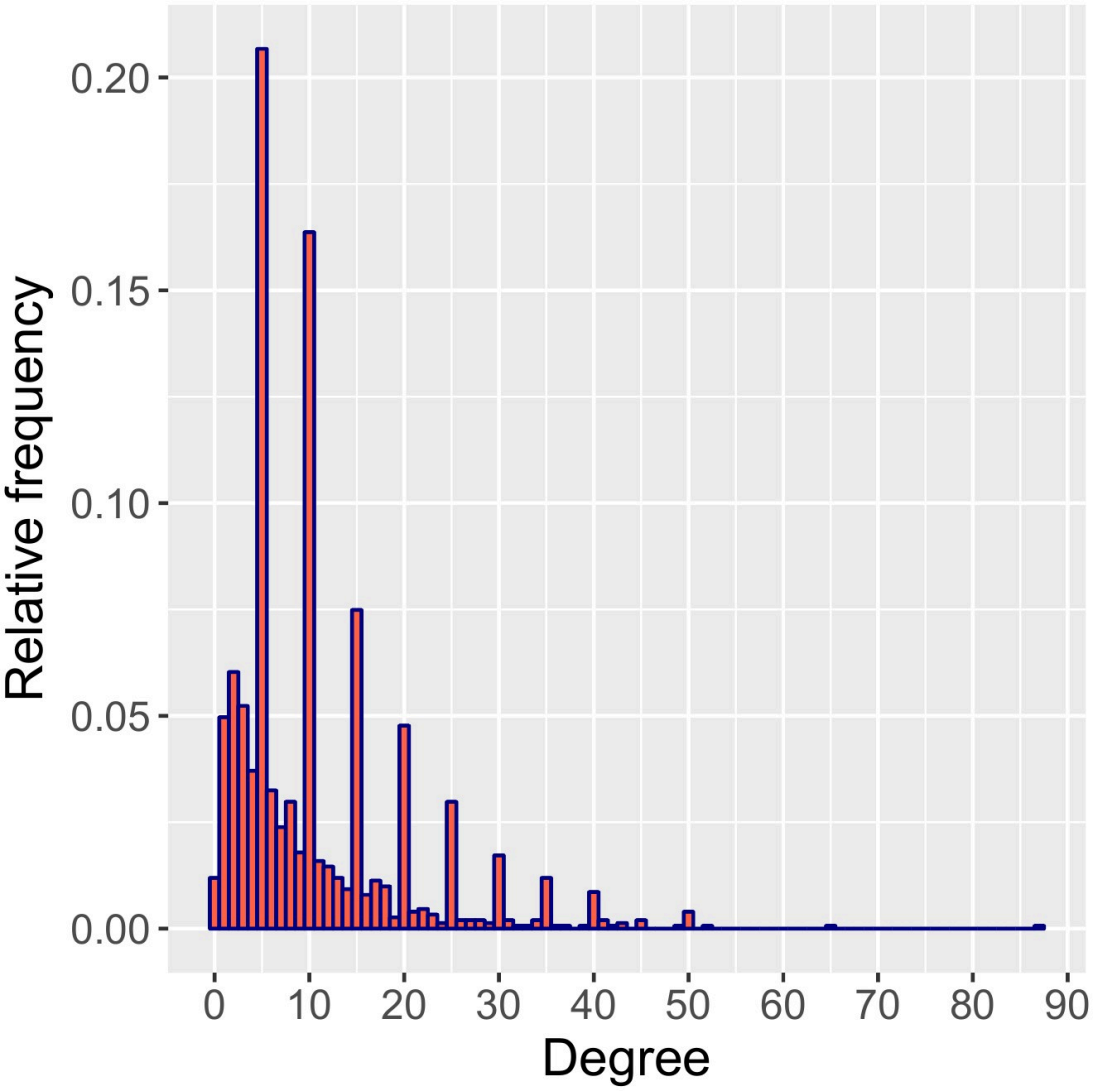
The observed degree distribution for the morning the day before the survey. The spikes result from rounding to multiples of five.

The survey asked respondents to round reported numbers of contacts to the nearest multiple of five, but not everyone did. The result is an observed degree distribution with spikes at multiples of five, but additional mass at other non-zero values as well (Fig 1). We create a mixture model to adjust for this feature of the data, which has been referred to as “heaping” or “coarsening” [52, 53]. For simplicity, we first describe our model for degrees for a single time interval, ensuring independent errors; we then expand our model to include multiple time intervals.

Let *C* denote the actual number of contacts, and let *Y* denote the reported number. We assume that the probability of rounding may differ when *C* < 5 and when *C* > 5, and that no positive numbers were rounded to zero. As such, we are assuming that respondents would be disinclined to report zero contacts when they actually made one or two. Define *α*_1_ = *P*(*Y* = 5|*C* ∈ {1, 2, 3, 4}), and define *α*_2_ to be the probability of rounding to the nearest multiple of 5 when *C* > 5. Then our assumptions imply that

$$\begin{array}{c} P(Y=0)=P(C=0) \end{array}$$

$$\begin{array}{c} P\left(Y=k\right)=\left(1-\alpha_{1}\right)P\left(C=k\right),\;\text{for}\;k\in \left\{1,2,3,4\right\} \end{array}$$

$$\begin{array}{c} P\left(Y=5\right)=\alpha_{1}\sum_{k=1}^{4}P\left(C=k\right)+P\left(C=5\right)+\alpha_{2}(P(C=6)+P(C=7)) \end{array}$$

$$\begin{array}{c} P\left(Y=k\right)=\left(1-\alpha_{2}\right)P\left(C=k\right), \end{array}$$

when *k* > 5 and k is not a multiple of 5, and

$$\begin{array}{c} P\left(Y=k\right)=P\left(C=k\right)+\alpha_{2}\sum_{k\in \{k-2,k-1,k+1,k+2\}}P\left(C=k\right), \end{array}$$

when *k* > 5 and k is a multiple of 5.

We assume that *C* follows a negative binomial distribution with unknown parameters *μ* and *r*. By substituting the negative binomial probability mass function into the above formulas, we obtain the likelihood function for the observed counts of contacts. We maximize the likelihood function using the optim function with the Nelder-Mead method in R to obtain maximum likelihood estimates for *α*_1_, *α*_2_, *μ*, and *r* [54].

We then expanded the negative binomial model to depend on covariates and include information from four different time intervals for each participant: the morning and afternoon/evening of the day before the survey and the morning and afternoon/evening of two days before the survey. To do so, we define the mean parameter to be a log-linear combination of covariate effects, including sex, categorized compound size, categorized age, symptom status, time of day (morning or afternoon/evening), and day relative to the survey day. Using 1_[condition]_ to represent an indicator variable taking the value 1 when a specified condition is met and 0 when not, we specify the relationship between the mean degree and predictors as follows:

$$\begin{array}{c} \text{log}\left(\mu \right)=\beta_{0}+\beta_{1}1_{\left[\text{symptomatic}\right]}+\beta_{2}1_{\left[6\leq \text{compound}\;\text{size}\leq 25\right]}\\ +\beta_{3}1_{\left[\text{compound}\;\text{size}>25\right]}+\beta_{4}1_{\left[\text{male}\right]}+\beta_{5}1_{\left[0\leq \text{age}\leq 5\right]}\\ +\beta_{6}1_{\left[6\leq \text{age}\leq 11\right]}+\beta_{7}1_{\left[12\leq \text{age}\leq 16\right]}\\ +\beta_{8}1_{\left[\text{Contact}\;\text{occurred}\;\text{in}\;\text{afternoon}/\text{evening}\right]}\\ +\beta_{9}1_{\left[\text{Contact}\;\text{occurred}\;\text{two}\;\text{days}\;\text{before}\;\text{survey}\right]} \end{array}$$ (1)

The maximum likelihood estimation approach is extended to estimate the covariate effects as well as the two rounding probabilities and the dispersion parameter. The error terms can no longer be assumed independent because we are including up to four degree observations per participant, so we fit robust “sandwich” standard errors. The jacobian function in the numDeriv package and the optim function in R (which optionally outputs the Hessian) were used to calculate the inner and outer matrices, respectively, for the sandwich variance [54, 55].

### 3.2 Multiple imputation for missing degree

The degree of an individual for a given time interval was calculated by summing the number of contacts reported in each location for all visited locations together with at-home contacts reported for that time interval. Each respondent was asked whether they had visited each of twelve locations, how many contacts were made there, and the time of the visit. If a respondent reported that she visited a location, but chose not to report the time or the number of contacts, then the degree is missing for that person. Since people visiting multiple locations are likely to make higher numbers of contacts outside the home, a complete-case analysis tends to underestimate numbers of contacts outside the home. This can be seen from Fig A in S2 Appendix. Furthermore, people who travelled outside the home tended to make higher numbers of contacts than those who stayed home (mean of 18.9 versus 8.8 on the morning before the survey day, for example), so the estimate of mean degree is biased downwards in a complete-case analysis.

We used multiple imputation to adjust for the bias in location distribution of contacts. The process imputes multiple missing variables before they are combined to calculate the degree for each individual. For outside-home locations, up to three variables may be missing: the response to “Was this location visited?”, the time of day (AM or PM) the location was visited, and the number of people contacted at that location. The responses to whether the location was visited were imputed based on a log binomial regression model with location type, symptom status, and age category as predictors, stratified on day relative to the survey day. Missing times were imputed by sampling from the distribution of non-missing times for that location type. To impute missing numbers of contacts for non-home locations, we fit a negative binomial distribution to the reported contact numbers, predicting the number contacted by the location, symptom status, time of day, and age category. For at-home contacts, we predicted number contacted based on symptom status, time of day, day relative to survey day, age category, and compound size. We are relying on the missing at random assumption that the covariates we are using to predict our imputed values are sufficient to explain differences between the observed and missing data [56]. Both types of reports were subject to the rounding issue noted above, so in both cases, the negative binomial model used for imputation was extended as described above to account for rounding. Otherwise, the imputed data would be smoother than the actual data and would bias our final estimates of the rounding probabilities downwards.

We created twenty imputed data sets by simulating from the predicted distributions and simulating the rounding process. We combined the point estimates and variances from the twenty data sets with MIcombine in the mitools package in R [57]. This package implements standard rules for multiple imputation to combine the twenty estimates and variances into a single estimate and variance for each parameter of interest [58]. The variance estimates using this technique combine uncertainty arising from the sampling process with that introduced by the imputation process. We report results based on the multiply imputed data, and we include our complete case analysis in S2 Appendix.

We then used the parameter estimates from our model to estimate the mean degree for the population. This was done separately for symptomatic and asymptomatic people, and separately for morning and afternoon. While the model coefficients in Eq 1 permit calculation of mean degree for any possible subgroup defined by the covariates, such mean degree values do not necessarily apply to the population of Niakhar because our sample was not representative. Therefore, we used a weighting procedure to adjust for the differences in age distribution between the survey sample and the Niakhar population. To do so, we first used the model coefficients to calculate the mean degree for all possible subgroups defined by combinations of the age, sex, and compound size categories. For example, the mean degree in the morning for asympomatic 0 − 5 year old females living in compounds with 6 − 25 members was calculated as $e^{\beta_{0}+\beta_{2}+\beta_{5}}$ based on the coefficients in Eq 1. Mean degrees for other subcategories were calculated similarly. Next, these were combined in a weighted average, where the weights reflect the age, sex, and compound size distributions of the Niakhar population. The estimates were calculated for the day before the survey day since the coefficient for two days before was nonsignificant. The mean degree estimates were calculated for each of the 20 imputed data sets, and variances were calculated by the delta method. The 20 estimates and variances were then combined using rules for multiple imputation with MIcombine [57].

### 3.3 Estimating compound homophily

*Homophily* is the tendency to contact others with similar characteristics [16], and has also been referred to as assortativity in the literature [59]. In this paper, we define homophily by compound membership to be the proportion of contacts which occur to one’s own compound members. While respondents did not report whether people they contacted were compound members or not, they did record numbers of contacts occurring in their own compound (i.e., at home).

We could estimate homophily by assuming that contacts within the respondent’s compound were exclusively to his own compound members. This would be assuming that (1) compound members are only contacted at home and (2) no one else is contacted at home. Yet 17% of morning and 14% of afternoon contacts occurred while the respondent was visiting another compound, likely to the residents of that compound, suggesting that the second assumption is too strong. By relaxing this assumption and by assuming that contacts reported in a visited compound include only members of the visited compound, we will show that homophily is estimated by the proportion of all contacts in the respondent’s compound minus the proportion of all contacts that occurred while the respondent was visiting another compound.

**Fig 2:**
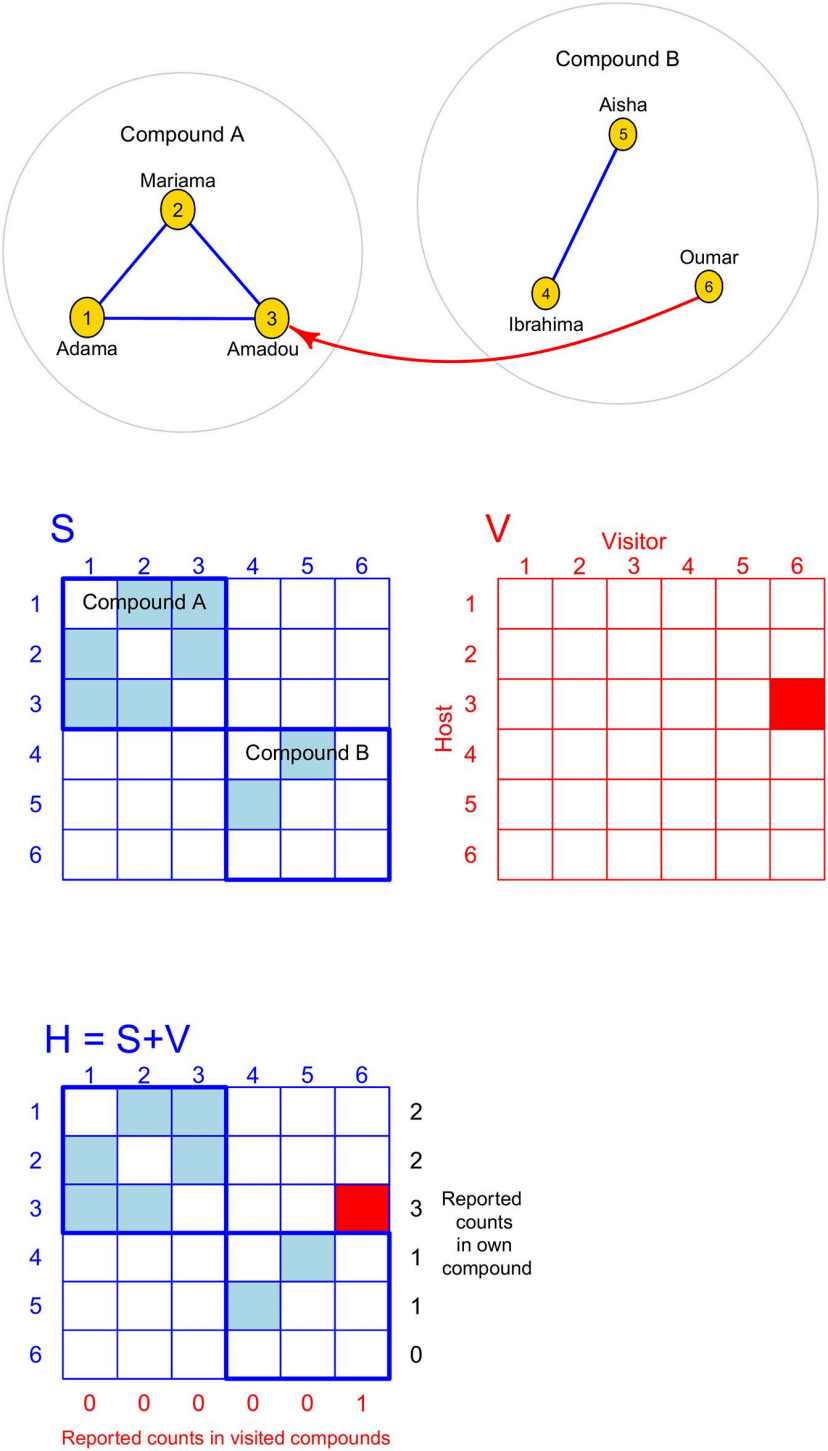
Toy example of a social network with adjacency matrices: *S* represents contacts between members of the same compound, *V* represents contacts between members of different compounds, and *H* = *S* + *V* has row sums equal to the numbers of contacts reported in the respondent’s compound.

To do so, we will visualize the social network as a graph where nodes depict social actors and edges represent contacts between them. A toy example, which we will use to walk the reader through our derivation, is given in Fig 2. Our example network includes two compounds, with contacts between compound members in blue and contacts between members of different compounds in red, with the arrow pointing from visitor to host. The arrow indicates that Oumar contacted Amadou while visiting Compound A. If the entire social network were observed, then our homophily value would be the number of edges connecting members of the same compound divided by the total number of edges in the network, $\frac{4}{5}$.

We will first demonstrate how our proposed estimator quantifies homophily when the entire network is sampled. In this case, the denominator for homophily—the number of edges—is calculated as the sum of degrees divided by two, since each edge is reported by two people. Letting *D*_*i*_ denote the degree of respondent *i*, the total number of edges is $\frac{\sum_{i=1}^{n}D_{i}}{2}$.

To calculate the numerator for homophily, we will define adjacency matrices to help express the relationship between the visualized network and the way the data were recorded. Let *S* denote an adjacency matrix (also called a sociomatrix) representing contacts between compound members: *S*_*ij*_ = 1 if *i* and *j* made contact and belong to the same compound and zero otherwise. *S* is symmetric because if *i* contacted *j*, then *j* also contacted *i*. The matrix *V* represents contacts between members of different compounds, and *V*_*ij*_ = 1 if *j* contacted *i* while *j* was visiting *i*’s compound. The asymmetry of *V* is used to distinguish the host from the visitor and will allow us to align this matrix with *S* to show the data captured by our survey. Here, *V*_3,6_ = 1 since persons 3 and 6 made contact while 3 was the host and 6 was the visitor. The elements of *S* and *V* were not collected in our survey since respondents did not record the exact identities of people they contacted. However, the column sums of *V* were collected as each respondent reported numbers of contacts made while visiting other compounds. The survey also collected numbers of contacts made within one’s own compound, which include those to one’s own compound members as well as to visitors. These reported counts are the row sums of *H* = *S* + *V*. What we are interested in for the numerator is the number of blue cells in the matrix *H*, since these represent within-compound contacts. We can obtain this by summing all elements of *H* and subtracting the red elements of *H*, and then dividing by two since each contact is reported twice—once by each person in the pair.

Let *V*_*i*_ denote the number of people person *i* reported contacting in other compounds—in other words, the sum of column *i* of the matrix *V*. Let *H*_*i*_ denote the number of people person *i* reported contacting at home—the sum of row *i* of *H*. If the entire network is observed, then the number of edges between members of the same compound is $\frac{\sum_{i=1}^{n}H_{i}-\sum_{i=1}^{n}V_{i}}{2}$. In our toy example, this is $\frac{9-1}{2}=4$, the numerator for our homophily value.

As noted above, the denominator for our homophily value is $\frac{\sum_{i=1}^{n}D_{i}}{2}$. Dividing numerator by denominator yields $\frac{\sum_{i=1}^{n}H_{i}-\sum_{i=1}^{n}V_{i}}{\sum_{i=1}^{n}D_{i}},$ which is the difference in proportion of contacts occurring in the respondent’s compound and the proportion of contacts occurring in visited compounds, as we set out to show.

We derived this method assuming the entire population was surveyed. In a random sampling scheme, we sum across randomly sampled rows of *H* and randomly sampled columns of *V*, and our estimator is unbiased. Since our sampling scheme favors symptomatic people, bias could be introduced if people are less likely to visit other compounds when they are symptomatic, but we found no evidence for this in the data (Fig D in S2 Appendix).

Violation of the two assumptions we have made tend to bias our homophily estimate downwards. First, some contacts made by visitors to other compounds may be to non-members. If this happens, these extra contacts would appear in *V* but not be included in the row sums of *H*. This means that too large a count would be subtracted from *H*, biasing the homophily estimate downwards. Next, some contacts between members of the same compound occur outside the compound. Our assumption that they do not would lead to an undercount of contacts between compound members, again leading to an underestimate of homophily.

Homophily was estimated separately for morning contacts by asympomatic people, afternoon/evening contacts by symptomatic people, morning contacts for symptomatic people, and afternoon/evening contacts for asymptomatic people using the multiply imputed data sets. Confidence intervals were calculated with a nonparametric bootstrap and combined with standard rules for multiple imputation by using MIcombine in the mitools package in R [57, 58].

### 3.4 Location distribution analysis

We created bar charts to display the location distribution of contacts using the multiply imputed data sets combined. To calculate confidence intervals for proportions of contacts within the respondent’s compound, we used an approach that applies a nonparametric bootstrap to multiply imputed data [60] as follows. The confidence interval for the proportion of contacts occurring in the respondent’s compound was calculated using a nonparametric bootstrap: for each imputed data set, 500 bootstrap resamples were drawn and proportions of contacts in the respondent’s compound were retained. The bootstrapped proportions were pooled across all imputations, and 2.5% and 97.5% quantiles were calculated for this pooled data set. Confidence intervals for proportions of contacts in other locations were calculated similarly. The location distribution of the original data is provided for comparison, as well as those stratified by age and by symptom status.

## 4 Results

A total of 6,758 surveys were collected from 5,557 participants. After restricting our analysis to a single survey per participant between August 1, 2009 to February 1, 2010, our data includes contact reports from 3,758 participants living in 345 compounds.

Table 1 summarizes the sex, ethnicity, and age distributions of participants analyzed in this paper and compares this to the distributions for participants who contributed to the complete-case analysis. More respondents were female (54%) than male (45.9%), and the majority were Serere (65.8%) or did not report their ethnicity (33.4%). The overall population of Niakhar at the start of the study was 51% female. Although we have access to ethnicity data only for contact survey respondents, Niakhar was 97% Serere in 2012, and our sample data is consistent with that [61]. The age distribution of our sample has more 0-5 year olds than the Niakhar population (60% as opposed to 24%) because this age group is particularly susceptible to acute respiratory infections. The proportion of 6-10 year olds in the sample is similar to that in the population (14.9% as compared to 16%) as was that for 12-16 year olds (5.1 as compared to 11%) but there were fewer adults in the sample than the population (19.9% instead of 49%).

**Table 1:** Distribution of sex, ethnicity, age, compound size, and symptom status for (1) all participants completing a survey between August 1, 2009 to February 1, 2010 and (2) all participants who contributed to the complete-case analysis.

Variable	All participants		Participants with non-missing degree	
	n	%	n	%
Sex				
Female	2031	54.0	878	51.6
Male	1724	45.9	824	48.4
Missing	3	0.1	0	0
Ethnicity				
Serere	2473	65.8	1158	68
Wolof	14	0.4	6	0.4
Other	16	0.4	3	0.2
Not Reported	1255	33.4	535	31.4
Age category				
0-5	2257	60.1	1169	68.7
6-11	560	14.9	215	12.6
12-16	193	5.1	66	3.9
>16	748	19.9	252	14.8
Symptoms, day before survey				
Asymptomatic	51	1.4	16	0.9
Symptomatic	3689	98.2	1686	99.1
Missing	18	0.5	0	0
Symptoms, two days before survey				
Asymptomatic	924	24.6	415	24.4
Symptomatic	2834	74.9	1287	75.6
Missing	0	0	0	0
Compound size				
1-5	61	1.6	32	1.9
6-25	1723	45.8	741	43.5
>25	1974	52.5	929	54.6
Median, Mean (SD)	27, 34.0 (31.7)		28, 37.5 (37.2)	

Only 61 of 3,758 respondents (1.6%) lived in compounds with five or fewer people, while 1,974 (52.5%) lived in compounds with over 25 people. Only 51 participants (1.4%) were asymptomatic on the day before the survey, but 924 (24.6%) were asymptomatic two days before the survey.

Among the 3,758 participants, 2,056 (54.7%) were missing either covariate values or values for variables which contributed to the calculation of the degree, such as the numbers of contacts made at home or the numbers of contacts made in a visited location. The distributions of covariates for participants who contributed to the complete-case degree analysis are shown in the two rightmost columns of the table. These distributions are similar to those of all analyzed participants, but a higher proportion of those included in the complete case analysis were 0-5 years old (68.7% versus 60.1%) and fewer were over 16 years old (14.8% versus 19.9%).

**Fig 3:**
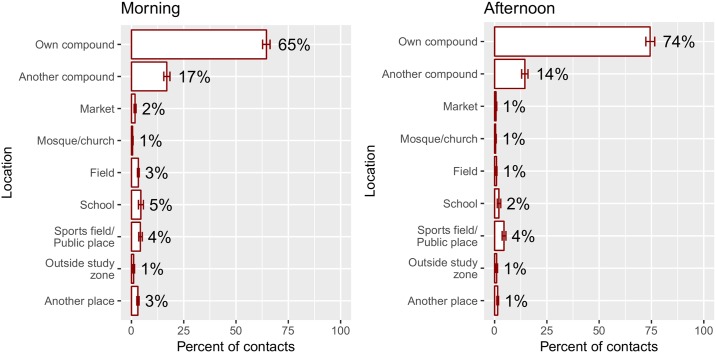
Percentage of contacts occurring in various locations using the multiply imputed data, two days before the survey day, with 95% confidence intervals.

Fig 3 shows the location distribution of contacts occurring two days prior to the survey by time of day using all multiply imputed data sets combined. The majority of contacts—65% in the morning and 74% in the afternoon/evening—occurred at home. The next most frequent location was in another compound.

School contacts comprised only 5% of morning contacts and 2% of evening contacts. This is likely because most respondents were not school-aged or did not attend school on the day of contact reports, because they were ill, because the day was a weekend day or holiday, or for some other reason. To better understand the behavior of school-aged children, we examined the location distribution of contact reports from children aged 6–16 years for weekdays between October and February. Contact reports from 121 symptomatic children met these criteria; 36% of such contacts occurred at school and 45% at home. Reports from 66 asymptomatic school-aged children met the criteria; for these, 27% of contacts occurred at school and 51% at home. As such, we observe higher proportions of contacts when we restrict our analysis to school-aged children on potential school days, but we do not know the proportion of children regularly attending school.

Location distributions were nearly identical between symptomatic and asymptomatic participants (Fig D in S2 Appendix). They were fairly similar across age categories, with the exceptions of 6-11 and 12-16 year olds making more school contacts than other age groups in the morning (10% and 18% respectively, Fig E in S2 Appendix), and adults making more at-home contacts in the evening (83% as compared to 70-75%; Fig F in S2 Appendix). Location distributions of contacts for the multiply imputed data are similar to those based on the original data, as evidenced by Figs B and C in S2 Appendix.

Table 2 summarizes missing data for variables that contributed to the calculation of the contact degree. The number of people contacted at home was missing for 53% and 55% of respondents for the morning and evening, respectively, on the day before the survey, and 60% and 61% of respondents for two days before the survey. Among participants who reported visiting at least one location outside the home, 47% and 46% did not report the number of people contacted in that location for the day before the survey and two days before, respectively. Similarly, 8% and 5%, respectively, were missing at least one time that a location was visited. In addition, 2% of participants had missing data for whether a location was visited or not, for at least one of the possible locations.

**Table 2:** Numbers and percentages of participants missing data contributing to the degree calculation.

Variable missing	Day before Survey		Two days before	
	n	%	n	%
Number contacted at home, AM	2003	53	2235	60
Number contacted at home, PM	2059	55	2276	61
For at least one outside location:				
Number contacted*	826	47	874	46
Time visited*	143	8	96	5
Whether visited	91	2	90	2

* Denominator is number of participants who visited at least one location.

Table 3 displays coefficient estimates with 95% confidence intervals for the degree distribution based on the multiply imputed data. The coefficient for “symptomatic” is 0.90, meaning that a symptomatic person makes 10% fewer contacts, on average, than an asymptomatic person (p = 0.006). The compound size and sex coefficients are not statistically significant. The age coefficients indicate that 0-5 year olds make 33% fewer contacts than people over 16 (p < 0.001), and 12-16 year olds make 13% more contacts than people over 16 (p = 0.016), controlling for other predictors.

**Table 3:** Estimates for coefficients, dispersion parameter, and rounding probabilities for the degree model.

Parameter	Estimate	95% Confidence Interval	P-value
Rounding probability, 1-4	0.28	[0.25, 0.31]	<0.001
Rounding probability, >5	0.52	[0.50, 0.53]	<0.001
Dispersion parameter	1.63	[1.57, 1.69]	<0.001
Model coefficient			
Intercept	15.58	[14.17, 16.98]	<0.001
Symptomatic (vs. asymptomatic)	0.90	[0.84, 0.95]	0.006
Compound size 6-25 (vs. ≤ 5)	1.05	[0.87, 1.23]	0.786
Compound size >25 (vs. ≤ 5)	1.17	[0.99, 1.35]	0.072
Male (vs. female)	1.03	[0.99, 1.06]	0.184
Age 0-5 years (vs. > 16)	0.67	[0.63, 0.71]	<0.001
Age 6-11 years (vs. > 16)	1.02	[0.96, 1.08]	0.626
Age 12-16 years (vs. > 16)	1.13	[1.04, 1.23]	0.016
Afternoon/evening (vs. morning)	0.91	[0.87, 0.94]	0.002
Two days before survey (vs. 1 day before)	1.04	[1.00, 1.07]	0.052

The mean number of contacts for afternoon/evening time points is 9% lower than for morning contacts (p = 0.002). The coefficient for “two days before the survey” is 1.04 and is not statistically significant (p = 0.052). Of course, the day relative to the survey day is an artifact of the data collection process; we added this predictor to assess potential recall bias. As this coefficient is greater than one (so is in the opposite direction that would indicate recall bias) and is not statistically significant, we do not have evidence for recall bias.

**Fig 4:**
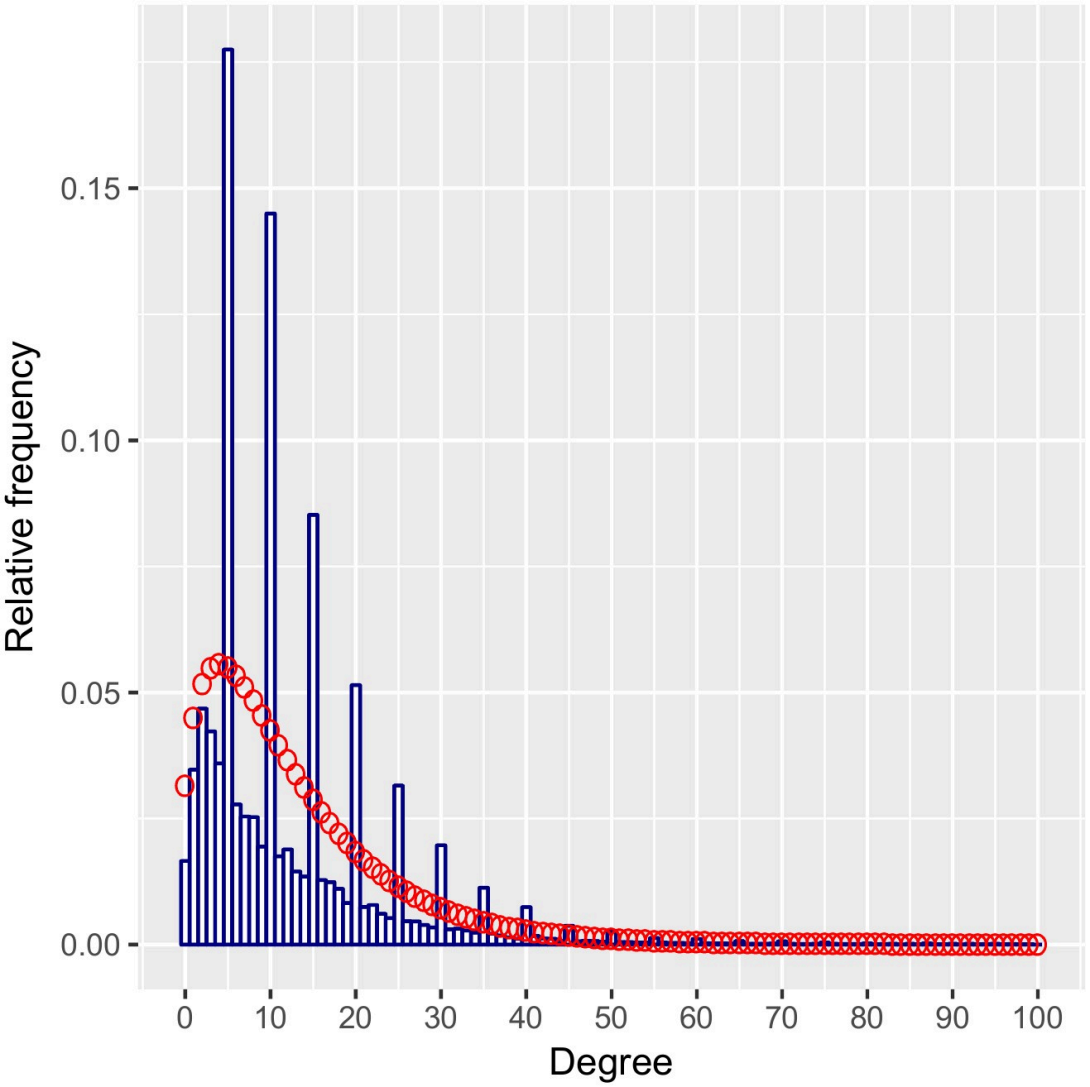
Histogram of multiply imputed distribution of reported degree overlaid with fitted underlying degree distribution.

**Fig 5:**
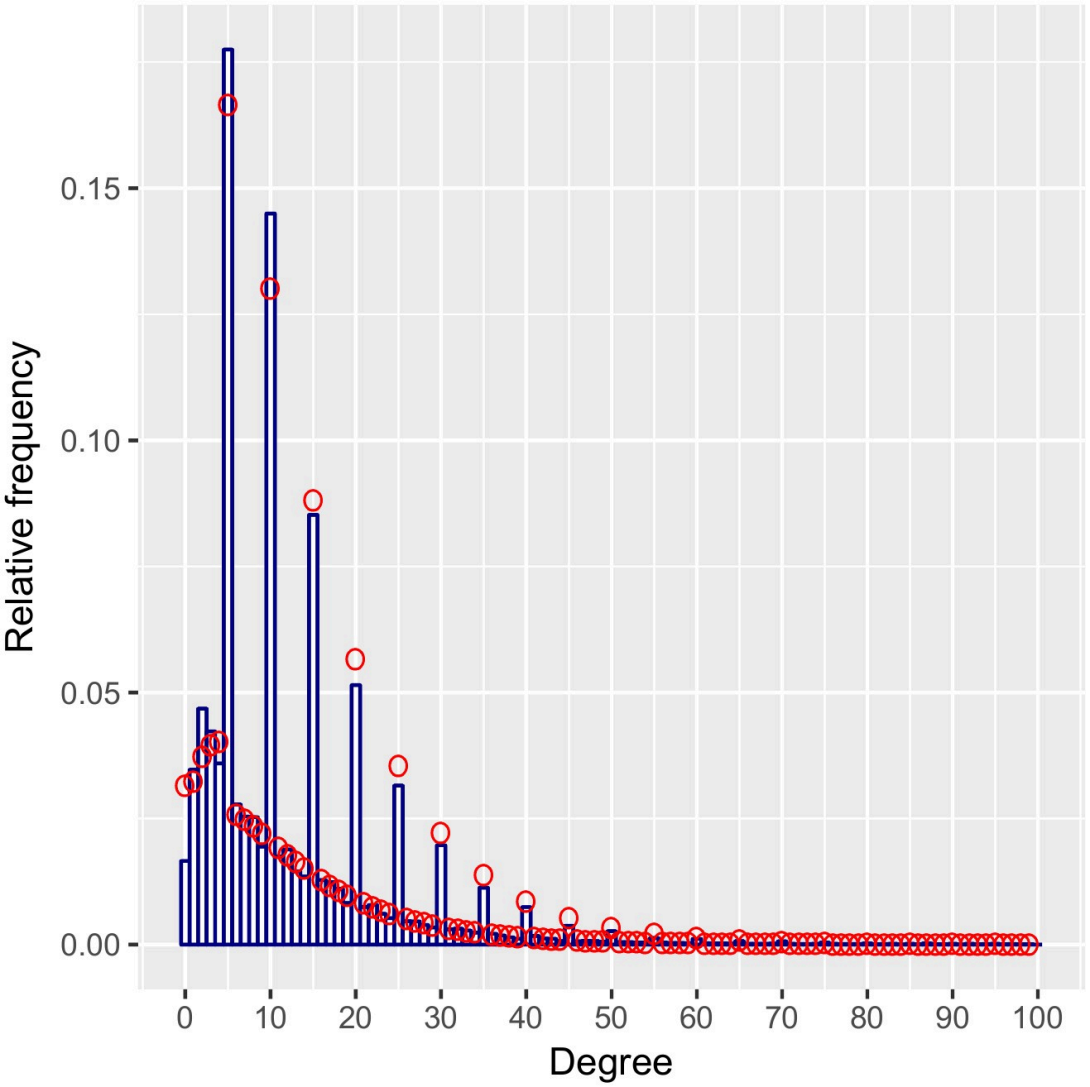
Histogram of multiply imputed distribution of reported degree overlaid with predicted distribution of contact reports inferred by the estimated underlying curve and estimated rounding probabilities.

As we had conjectured, numbers of contacts between one and four are less likely to be rounded. We estimate the probability of rounding to be 0.28 when the true degree is between one and four, and 0.52 when the true degree is greater than five. The rounding probability for contacts >5 does not correspond exactly to the respondent rounding probability since these degrees are based on the sum of multiple contributing variables. However, the rounding probabilities allow us to graphically assess whether our negative binomial model is an appropriate model based on the observed data. The degree distribution for the multiply imputed data is compared to the inferred underlying distribution in Fig 4. Our model allows us to visualize what the actual degree distribution in this population is, without the heaping, which arises as an artifact of reporting. However, because the inferred distribution is qualitatively different from the data, it is difficult to assess goodness-of-fit from this graph. To do so, we calculated the inferred distribution of reported contacts by combining our estimated rounding probabilities with our inferred negative binomial probabilities using the equations in Section 3.1. Thus we obtain an inferred distribution of reported contacts. We compare this to the empirical distribution of reported contacts in Fig 5. Our predictions fit the observed reports quite well though we may be overestimating the number of reported zeroes. We have truncated the x-axis at 100 since only 0.16% of imputed degrees were over 100 while our model places only 0.01% of probability mass on that range. As such our model underestimates the chance of extreme values in the tail.

Table 4 displays inferred mean degree values for the population of Niakhar. The mean degree in the morning is 16.5 for asymptomatic people and 15.0 for symptomatic people. Mean degrees in the afternoon are 14.8 and 13.5 for asymptomatic and symptomatic people, respectively. The overlap in confidence intervals indicates that the mean degrees for the different time points and symptom statuses are not significantly different.

**Table 4:** Inferred mean degree by symptom status and time point.

Symptom Status	Time Point	Mean Degree	95% Confidence Interval
Asymptomatic	AM	16.5	[14.3, 18.7]
Asymptomatic	PM	14.8	[12.7, 16.9]
Symptomatic	AM	15.0	[12.9, 17.1]
Symptomatic	PM	13.5	[11.5, 15.4]

Table 5 shows our homophily estimates (the proportion of contacts occurring to one’s own compound members), separately by symptom status and time of day. These are, in general, lower than the proportion of contacts occurring in one’s own compound since a subset of those occurred to visitors. A higher proportion of afternoon/evening contacts are to one’s own compound members than morning contacts (60.1% versus 48.2% for symptomatic people and 59.1% vs 46.0% for asymptomatic people).

**Table 5:** *Compound homophily estimates*: Estimated proportion of contacts to own compound members by symptom status and time of day, two days before survey.

Time of Day	Symptomatic		Asymptomatic	
	Percent	95% C.I.	Percent	95% C.I.
Morning	48.2	[44.8, 51.6]	46.0	[40.8, 51.3]
Afternoon/evening	60.1	[56.2, 64.1]	59.1	[52.7, 65.5]

Symptomatic people make slightly higher proportions of contacts to their own compound members than asymptomatic people at both time points, but the slight differences are not statistically significant as indicated by the substantial overlap in confidence intervals.

## 5 Discussion

We have summarized the degree distribution, homophily by compound membership, and location distribution of face-to-face social contacts in Niakhar, Senegal, based on two consecutive days of survey reports from 3,758 participants over a period of 6 months.

For asymptomatic Niakhar residents, we estimated a mean contact degree of 16.5 (95% C.I. 14.3, 18.7) in the morning and 14.8 in the afternoon (95% C.I. 12.7, 16.9). While the extent of overlap between morning and afternoon contacts is not known, 16.5 is a lower bound on the degree for the entire day (assuming maximal overlap); the actual mean degree for the entire day is probably higher. This estimate places this rural Senegalese community at the high end when ranking cultures based on contact degree. The mean number of contacts in the multi-country POLYMOD study was 7.9 for Germany, between 11 and 12 for Belgium, Finland, and Great Britain, 17.5 for Luxembourg, and 19.8 for Italy [17]. A recent analysis of diary-based contact surveys in Zimbabwe found people made 11.1 contacts per day on average, and the average was higher in their peri-urban site than their rural site (11.6 versus 10.8) [30]. A large survey in coastal Kenya estimated a mean of 17.7 contacts per person per day; this was higher for residents of rural areas than for those in semiurban areas (18.8 versus 16.5) [31]. A household-based survey of travel and contact patterns in Guangdong, China, found that the mean numbers of contacts per day ranged from 17–22 for those under the age of 60 [35].

Our findings that people aged 0-5 years make fewer contacts than adults and that sex and household size are not significantly related to numbers of contacts are consistent with results from other countries [17, 31, 33]. However, a study of French contact patterns found significantly more contacts by females than males and significantly fewer contacts by those in small households [29]. The apparent differences between the French study and the results we report here may be, in part, attributable to cultural difference between France and Senegal, and, in part, due to how each study categorized household size (they categorized as 1, 2, 3, 4, and ≥ 5 while we used the 0-5, 6-25, and > 25 as our categories). In addition, the distribution of household sizes is very different in the two settings with much larger households in Senegal.

We estimate that the majority of contacts occur in the home in this community: 65% of morning contacts and 74% of afternoon/evening contacts. This is similar to results from a diary-based survey of social contacts in Vietnam, which found that the majority (85%) of contacts occurred at home, followed by school (5%) and workplace (4%) [33]. However, this contrasts with estimates for eight European countries in the POLYMOD study, which found that 20-30% of contacts occurred at home within each country, with similar proportions occurring at work or in leisure activities and fewer occurring in schools [17].

We found that symptomatic people make significantly fewer contacts than asymptomatic people, but the reduction was small in size. The average number of contacts was 16.5 in the morning for asymptomatic people. It was 15.0 for symptomatic people at this time point, but these values are not significantly different (Table 4). Researchers analyzing data from England [62] also found a reduction but a much larger one; their averages were 14.9 for healthy people and 3.8 for sick people. This could be due to cultural differences in withdrawing from work or school while ill, higher numbers of people working within the home in Senegal, and/or to the limited representation of school contacts in our data set.

We quantified compound homophily by estimating that 48.2% and 60.1% of contacts by symptomatic people occur to their own compound members in the morning and afternoon/evening respectively, with nearly identical (though slightly smaller) estimates for asymptomatic people. This is consistent with epidemic models estimating higher transmission rates between household members than non-members [63–65]. Other contact surveys we reviewed did not estimate household homophily, but instead estimated age-based mixing patterns. Our design did not allow for estimating age-based mixing, and we would recommend that future surveys record the age of people contacted in order to allow for this.

Some of the limitations of our data may guide future researchers in survey design. One limitation was the large amount of missing data in our study. While we used multiple imputation to reduce bias from this shortcoming, we relied on the assumption that the data were missing at random. As our imputation process includes key covariates found to predict contact patterns in other studies, we believe our assumption is reasonable. However, because this assumption can never be validated [66], it would be far better to have a more complete data set. To reduce missing data, we recommend consideration of a diary-based approach when literacy is at sufficient levels. When implementing a diary survey in a setting without high literacy, we recommend the methods used in a study in coastal Kenya [31]. These researchers employed focus group interviews and a small pilot study prior to survey implementation, which helped participants understand the purpose and methods of the survey. They selected a “shadow” respondent for participants under 10 and those who were illiterate, and they provided participants with wrist watches programmed to sound an alarm every hour to remind them to update their diary.

Another limitation is the limited data on school contacts collected in our study. This partly because some contact interviews took place on days that were not school days. Additionally, some respondents may not have attended school regularly or may not have attended on the survey day even when it was a school day. We recommend designing surveys to record school attendance patterns and whether the survey day is a school day or holiday.

Collecting contact reports on the day of the survey, when many surveys were conducted in the morning, induced truncation bias for those reports. Recording the time of the interview would provide the researcher information to adjust for this, but assumptions would need to be made regarding the distribution of contacts over time. It would be cleaner and save resources to not record contacts that took place on the day of the survey. As no evidence for recall bias was found, this approach would likely collect sufficient information.

Our focus on symptomatic participants was less of a limitation than anticipated, since a fair amount of data on asymptomatic contact reports was nonetheless collected, allowing us to estimate differences in contact patterns based on symptom status, and because we did not find large differences based on symptom status. The age composition of our sample differed from that of the overall population due to the higher frequency of febrile respiratory illness among young children. We recommend random sampling to ensure that contact patterns are measured in a representative way.

While we had not explicitly asked participants which contacts were to household members, we devised an estimator to quantify household homophily. Our assumptions that household members are not contacted outside the home and that contact counts for visited compounds only include members of the visited compound are quite strong, and their violation results in an underestimate of homophily. In future surveys, it would be preferable to solicit numbers of contacts to household members, as these groups are critical for disease transmission.

The estimates presented in this paper may be used to perform epidemic simulations for the population of Niakhar. To do so, the population may be visualized as a set of nodes, which may be connected by edges defining their contacts. The morning degree distribution for the set of nodes would be simulated from the using the parameters in Table 3. Next, degrees between compound members would be randomly linked until the required compound homophily level is attained. School structure could be added by linking a subset of the remaining unmatched degrees between children of the same age, based on school enrollment and attendance data. Finally, the remaining unlinked degrees would be randomly linked between village members. This would form one instance of the contact network. Next an SEIR (Susceptible-Exposed-Infected-Removed) process may be simulated over the contact network by first randomly selecting a subset of residents to be infected as “seeds” for the epidemic. The disease may be transmitted over edges connecting infected and susceptible people with age-specific transmission probabilities. Those infected move into the exposed state, then to the infected state, then the recovered state, based on prefined probability distributions for the pathogen of interest. The process would be repeated for the afternoon time point and would continue until all residents are either infected or recovered. The simulation would then be repeated thousands of times, tracking epidemic outcomes over the simulations such as the proportion infected, the epidemic peak date, and others. The impact of social distancing strategies on epidemic outcomes could also be investigated. For example, a strategy of quarantining residents to their compound when they manifest symptoms could be simulated by deleting all non-compound contacts for those who show symptoms until they move to the recovered state.

We have provided the first analysis of face-to-face contact network data in rural Senegal and quantified key network structures: heterogeneity in degree based on individual attributes, homophily by compound membership, and the location distribution of contacts. By using a latent variable model to account for irregular rounding of contact reports, we were able to validate the fit of our model to the degree distribution. Our estimated parameters may be used as input into epidemic simulation models for cross-cultural comparisons. As our mean degree is at the high end of the range for various cultures and contexts, one might expect rural Senegal to fall at the higher end when it comes to epidemic outcomes that depend on these types of contacts for transmission. However, the limited impact of symptom status on contact patterns is also a key factor. We expect a disease would spread farther and faster than in a culture with similar degree distribution but stronger social distancing in response to respiratory illness. Such a comparison may be undertaken as future work.

## Supporting information

S1 Appendix Social contact survey. The form used to conduct face-to-face interviews regarding the respondent’s social contact patterns.
(PDF)

S2 Appendix Supplementary analyses and data cleaning. This appendix summarizes analyses performed on the complete-case data set, compares them to results based on the imputed data set, and also presents stratified analyses of the location distribution of contacts. The appendix also includes a description of data cleaning that was performed.
(PDF)
