# Supplementary material for: Networks of face-to-face social contacts in Niakhar, Senegal
Source: PLoS One. 2019 Aug 6;14(8):e0220443. doi: 10.1371/journal.pone.0220443 (PMC6684077; doi:10.1371/journal.pone.0220443)
Supplement: S1 Appendix — The form used to conduct face-to-face interviews regarding the respondent’s social contact patterns. (PDF) [file pone.0220443.s001.pdf]

Évaluation de l'efficacité d'un vaccin antigrippal trivalent saisonnier chez les enfants sénégalais	Community Contact Form<br>The location of cases during their infectious period	Form **J** v. 1

Community Contact Form

The location of cases during their infectious period

Subject's DSS Number: |__|__|__|__|__|__|__|__| **Interview Time: AM** ☐ **PM** ☐

Onset date of the first influenza symptom: |__|__| / |__|__| / |__|__| (DD / MM / YY)

Today

No. of individuals the subject spoke with in his/her compound **AM:** |__|__| **PM:** |__|__|

Location	Did the subject visit any of these locations? Yes	No	When did the subject visit? AM	PM	No. of individuals the subject spoke with	No. Village	No. Compound
**Another Compound**							
1	☐	☐	☐	☐	|__|__|	|__|__|	|__|__|__|
2	☐	☐	☐	☐	|__|__|	|__|__|	|__|__|__|
3	☐	☐	☐	☐	|__|__|	|__|__|	|__|__|__|
4	☐	☐	☐	☐	|__|__|	|__|__|	|__|__|__|
5	☐	☐	☐	☐	|__|__|	|__|__|	|__|__|__|
Market	☐	☐	☐	☐	|__|__|	|__|__|	
Mosque / Church	☐	☐	☐	☐	|__|__|	|__|__|	
Field	☐	☐	☐	☐	|__|__|	|__|__|	
School	☐	☐	☐	☐	|__|__|	|__|__|	
Sports field / Public place	☐	☐	☐	☐	|__|__|	|__|__|	
Outside of the study zone	☐	☐	☐	☐	|__|__|	Specify : ______________	
Another place:	☐	☐	☐	☐	|__|__|	|__|__|	

Specify: ______________________________

Yesterday

No. of individuals the subject spoke with in his/her compound **AM:** |__|__| **PM:** |__|__|

Location	Did the subject visit any of these locations? Yes	No	When did the subject visit? AM	PM	No. of individuals the subject spoke with	No. Village	No. Compound
**Another Compound**							
1	☐	☐	☐	☐	|__|__|	|__|__|	|__|__|__|
2	☐	☐	☐	☐	|__|__|	|__|__|	|__|__|__|
3	☐	☐	☐	☐	|__|__|	|__|__|	|__|__|__|
4	☐	☐	☐	☐	|__|__|	|__|__|	|__|__|__|
5	☐	☐	☐	☐	|__|__|	|__|__|	|__|__|__|
Market	☐	☐	☐	☐	|__|__|	|__|__|	
Mosque / Church	☐	☐	☐	☐	|__|__|	|__|__|	
Field	☐	☐	☐	☐	|__|__|	|__|__|	
School	☐	☐	☐	☐	|__|__|	|__|__|	
Sports field / Public place	☐	☐	☐	☐	|__|__|	|__|__|	
Outside of the study zone	☐	☐	☐	☐	|__|__|	Specify: ______________	
Another place:	☐	☐	☐	☐	|__|__|	|__|__|	

Specify: ______________________________

Day before yesterday

No. of individuals the subject spoke with in his/her compound **AM:** |__|__| **PM:** |__|__|

Location	Did the subject visit any of these locations? Yes	No	When did the subject visit? AM	PM	No. of individuals the subject spoke with	No. Village	No. Compound
**Another Compound**							
1	☐	☐	☐	☐	|__|__|	|__|__|	|__|__|__|
2	☐	☐	☐	☐	|__|__|	|__|__|	|__|__|__|
3	☐	☐	☐	☐	|__|__|	|__|__|	|__|__|__|
4	☐	☐	☐	☐	|__|__|	|__|__|	|__|__|__|
5	☐	☐	☐	☐	|__|__|	|__|__|	|__|__|__|
Market	☐	☐	☐	☐	|__|__|	|__|__|	
Mosque / Church	☐	☐	☐	☐	|__|__|	|__|__|	
Field	☐	☐	☐	☐	|__|__|	|__|__|	
School	☐	☐	☐	☐	|__|__|	|__|__|	
Sports field / Public place	☐	☐	☐	☐	|__|__|	|__|__|	
Outside of the study zone	☐	☐	☐	☐	|__|__|	Specify: ______________	
Another place:	☐	☐	☐	☐	|__|__|	|__|__|	

Specify: ______________________________

Did the subject visit another location, not specified above, during the last 7 days?

Yes: ☐ **--> Specify:**

No: ☐ **No. Village:** |__|__| **Details:** ______________________________

Signature of the interviewer:______________________________ **Interviewer No.: TIV –** |__|__|__|–|__|__|__|
