# Supplementary material for: Networks of face-to-face social contacts in Niakhar, Senegal
Source: PLoS One. 2019 Aug 6;14(8):e0220443. doi: 10.1371/journal.pone.0220443 (PMC6684077; doi:10.1371/journal.pone.0220443)
Supplement: S2 Appendix — This appendix summarizes analyses performed on the complete-case data set, compares them to results based on the imputed data set, and also presents stratified analyses of the location distribution of contacts. The appendix also includes a description of data cleaning that was performed. (PDF) [file pone.0220443.s002.pdf]

Appendix 2

This appendix includes supplementary data analyses in section 1 and a description of the data cleaning procedure in section 2.

1 Supplementary Data Analyses

This section summarizes analyses performed on the complete-case data set, compares them to results based on the imputed data set, and also presents stratified analyses of the location distribution of contacts.

Fig A compares the location distribution of contacts two days prior to the survey by time of day between participants contributing to the complete case analysis to that for all participants who have at least some location data. There are fewer outside-home contacts for participants contributing to the complete-case analysis since participants were excluded from this analysis if they missed one or more responses to number of contacts at any visited location and because those who travelled tended to make higher numbers of contacts.

Figs B and C compare the location distributions of contacts between the original data and imputed data. The slight differences arise from differences in covariates which are predictors in the imputation process.

Fig D shows nearly identical location distributions of contacts between symptomatic and asymptomatic participants, and Fig E and Fig F show slight differences in location distribution by age category. These figures were created using the imputed data as we expect that to represent the actual distribution of contacts more accurately.

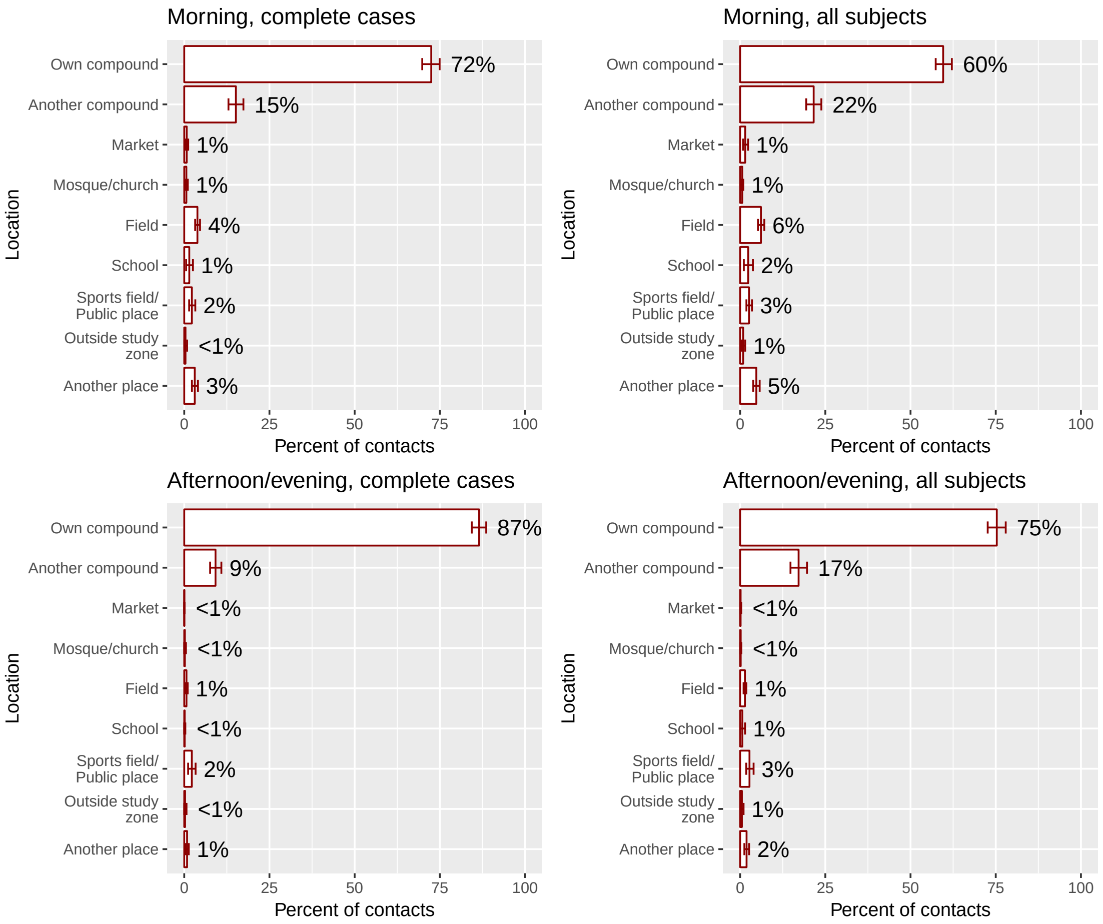

Figure A: Comparison of location distribution of contacts reported by complete cases for the degree analysis (left), to that by all participants who have at least one report for one location (right), two days before the survey day, with 95% confidence intervals.

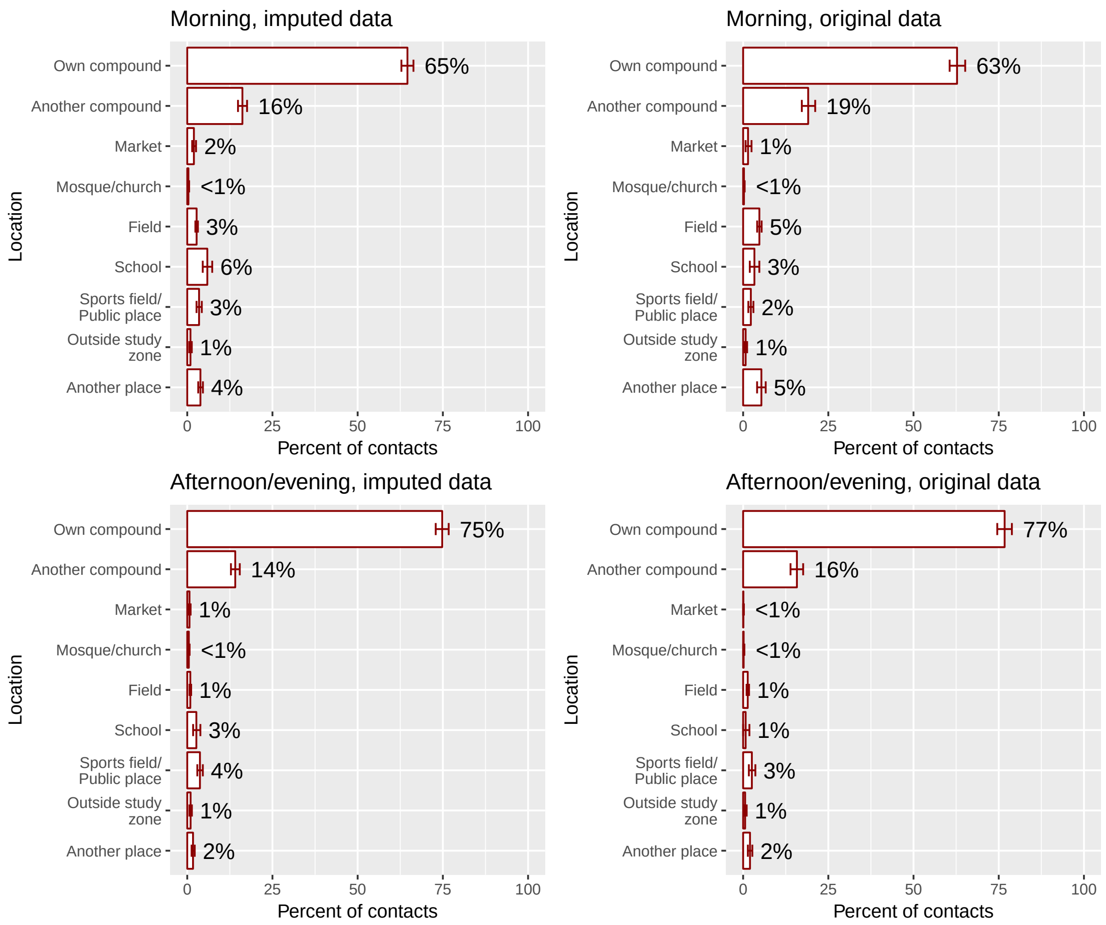

Figure B: Comparison of location distribution for the imputed and original data, one day before the survey, with 95% confidence intervals.

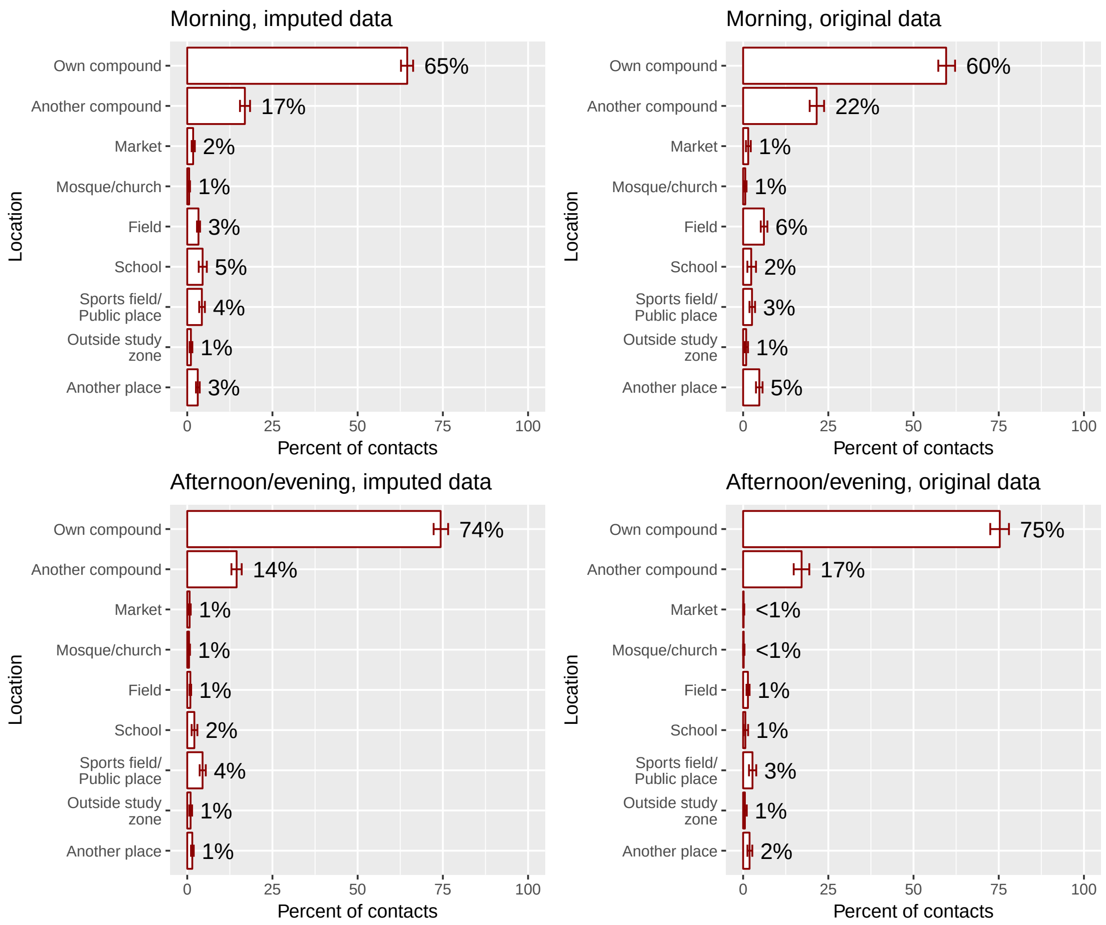

Figure C: Comparison of location distribution for the imputed and original data, two days before the survey, with 95% confidence intervals.

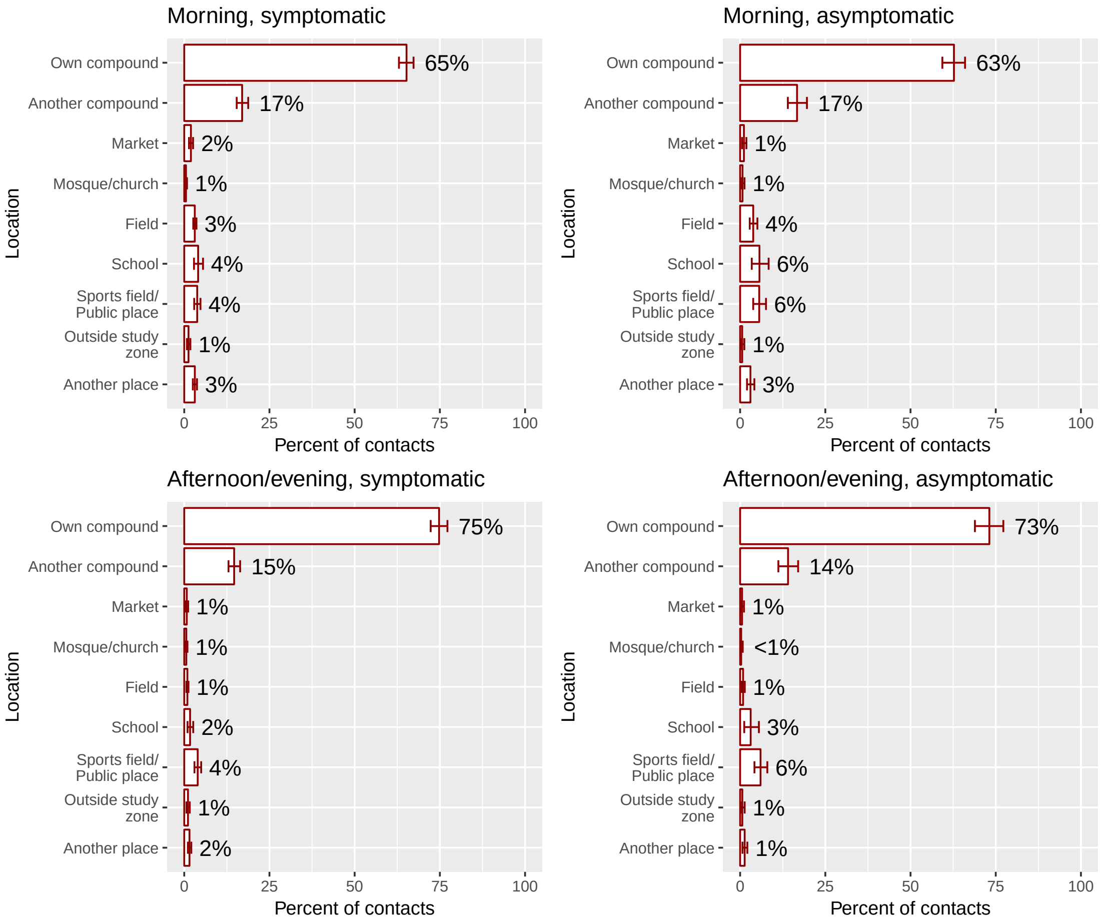

Figure D: Comparison of location distribution between symptomatic and asymptomatic participants, two days before the survey day, with 95% confidence intervals.

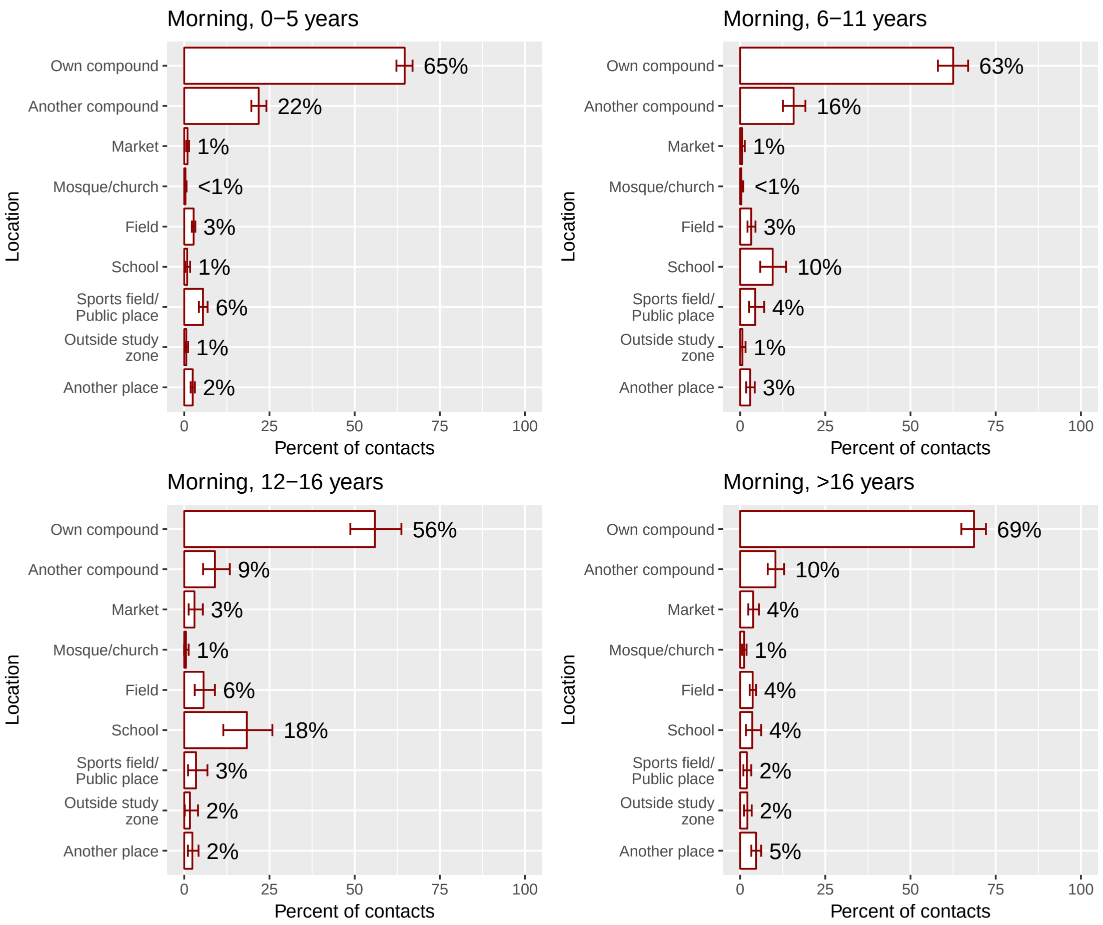

Figure E: Comparison of location distribution between age groups in the morning, two days before the survey day, with 95% confidence intervals.

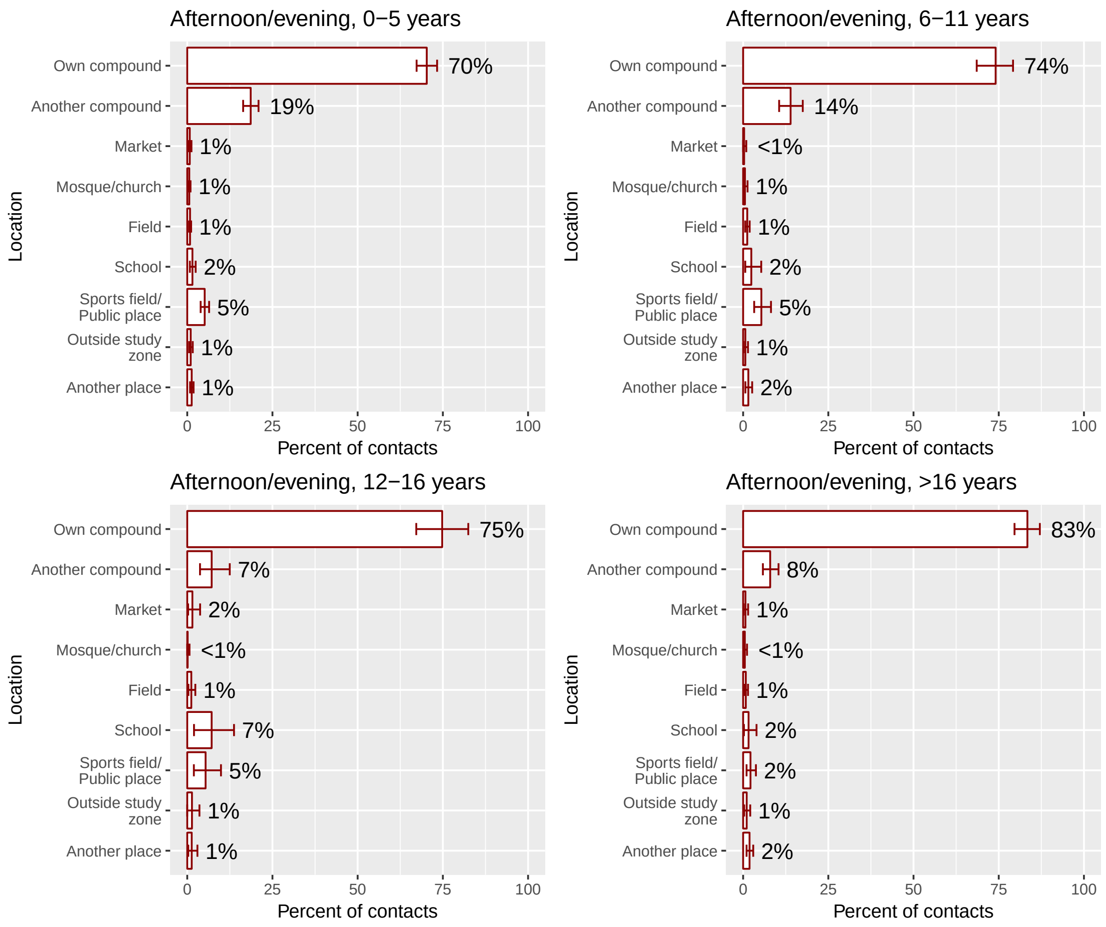

Figure F: Comparison of location distribution between age groups in the afternoon/evening, two days before the survey day, with 95% confidence intervals.

Table A shows mean degree for the original data as well as for one imputed data set. The imputed degrees are higher, as we may expect. This is because (as noted the main text), respondents in need of imputation tended to travel to more locations outside the home, and those who travelled tended to make higher numbers of contacts than those who stayed home. Therefore the estimate of mean degree is biased downwards in a complete-case analysis.

Table A: Comparison of mean degree between original and imputed data.

Time point	Original data	Imputed data
Day before survey AM	10.8	13.1
Day before survey PM	9.7	11.8
Two days before survey AM	11.7	13.9
Two days before PM	10.4	12.6

Table B shows coefficient estimates for the complete-case analysis of the degree distribution. The rounding probability for degree between 1 and 4 is similar to that estimated with the imputed data set (0.28). The rounding probability for contacts > 5 is higher because in the original data, degree tended to be summed across a smaller number of variables due to the large amount of missing data for outside-home contacts. Therefore, fewer calculated degree values were the sum of a rounded and a non-rounded degree in this data set. Other coefficient estimates are fairly similar to the estimates based on the imputed data. The exceptions are the coefficient for the 6-11 year old group (less than one and is statistically significant in this model; not significant in the other), and the 12-16 year old group (> 1 and significant in this model, not significant in the other). These differences are due to differences in age composition between the complete-case data set and the full data set, as well as differences in age composition between respondents missing specific location variables.

Table B: Coefficient estimates for negative binomial model of contact degree, complete case analysis.

Parameter	Estimate	95% Confidence Interval	P-value
Rounding probability, 1-4	0.24	[0.21, 0.27]	<0.001
Rounding probability, >5	0.64	[0.62, 0.65]	<0.001
Dispersion parameter	1.79	[1.70, 1.89]	<0.001
Intercept	15.84	[15.5, 16.18]	<0.001
Symptomatic	0.90	[0.83, 0.98]	0.01
Compound size 6-25	0.94	[0.62, 1.26]	0.734
Compound size >25	1.16	[0.84, 1.48]	0.334
Male	0.98	[0.93, 1.03]	0.426
Age 0-5	0.58	[0.52, 0.64]	<0.001
Age 6-11	0.91	[0.83, 0.99]	0.021
Age 12-16	0.99	[0.89, 1.10]	0.885
Afternoon/evening	0.94	[0.90, 0.99]	0.026
Two days before survey	0.98	[0.93, 1.03]	0.503

Fig G compares the actual degree distribution to the underlying fitted distribution. The underlying fitted distribution does not account for the rounding process which was an artifact of data collection, so is more representative of the actual degree distribution. The proportion of observed degrees greater than 70 was 0.16%, while the probability mass placed by our model on that interval is 0.004%, so the model underestimates mass in the tail of the distribution. The highest observed degree was 140. To further assess goodness-of-fit, we combined the inferred negative binomial distribution with our estimated rounding probabilities to obtain an inferred probability distribution for numbers of contact reports. This is compared to the empirical distribution of contact reports in Fig H.

Table C presents homophily estimates based on the complete-case data set. These show higher levels of homophily than in our multiple imputation analysis, as expected.

Table C: Homophily estimates: Estimated proportion of contacts to own compound members by symptom status and time of day, two days before survey, complete case analysis.

	Symptom atic		Asymptomatic	
Time of Day	Percent	95% C.I.	Percent	95% C.I.
Morning	58.1	[53.5, 62.6]	54.9	[44.6, 63.9]
Afternoon/evening	78.5	[74.1, 82.4]	73.6	[65.7, 81.1]

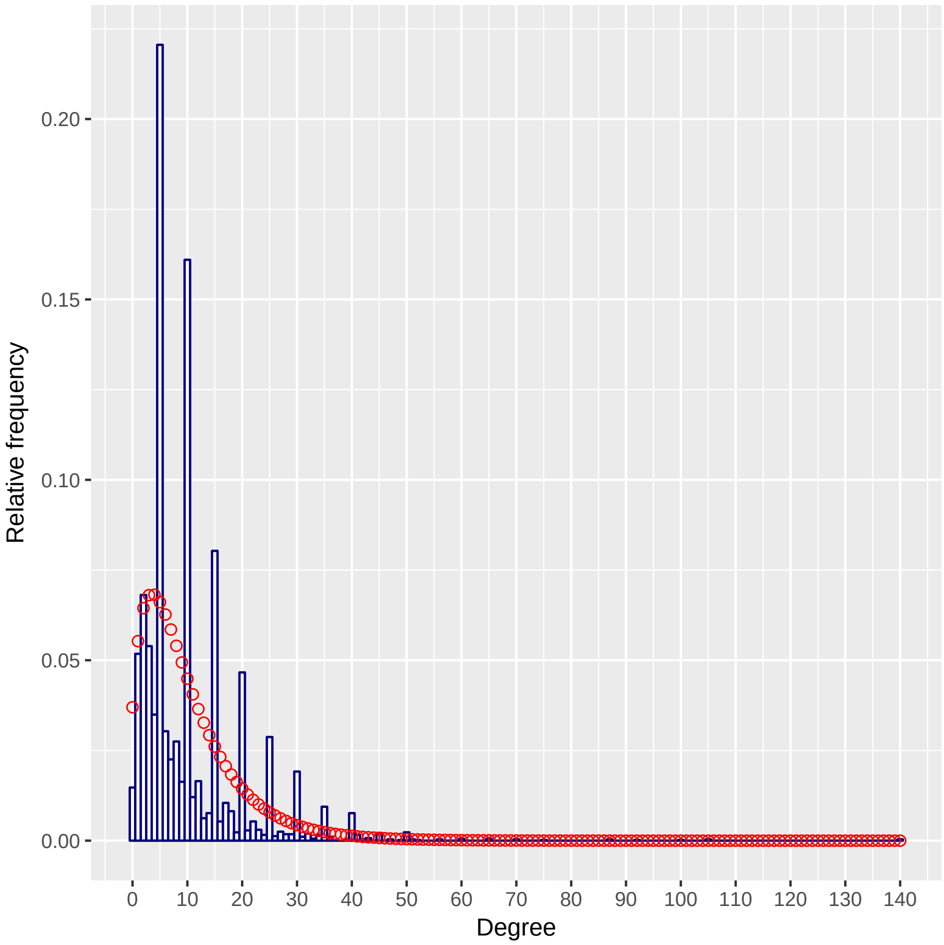

Figure G: Observed degree distribution with fitted underlying degree distribution, complete case analysis.

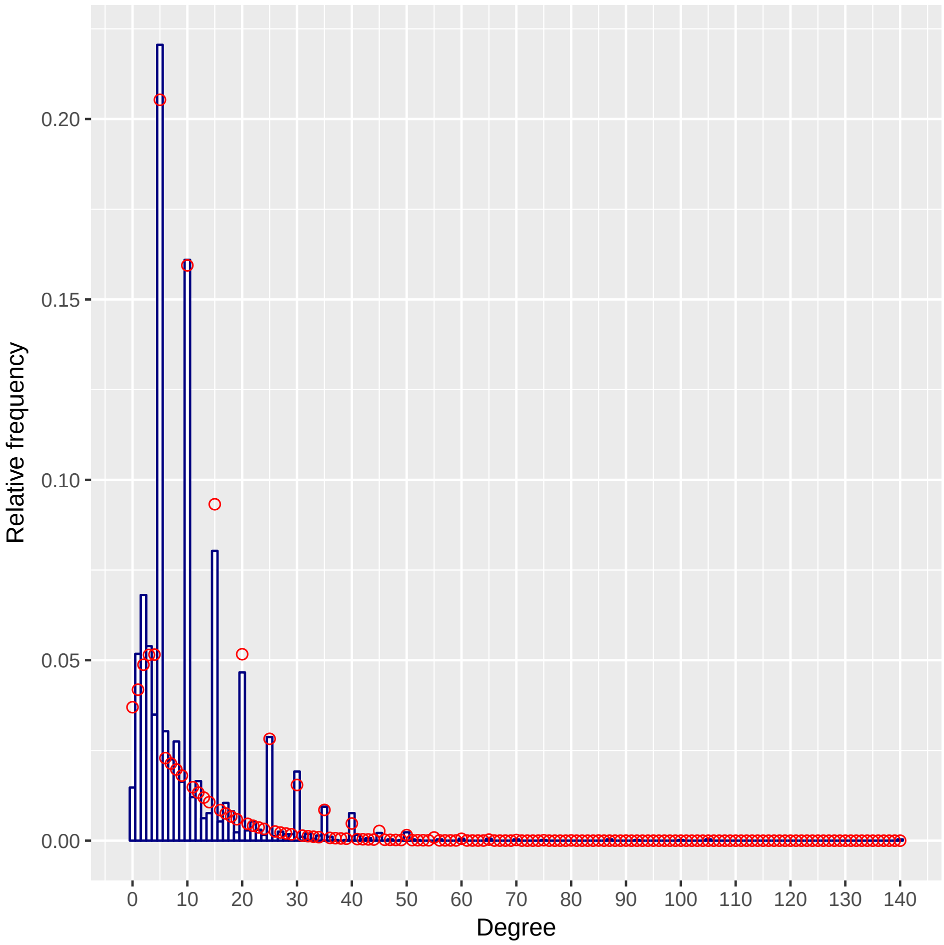

Figure H: Observed degree distribution with predicted distribution of contact reports, complete case analysis

2 Data cleaning

This section summarizes data cleaning that was performed programmatically after all data were collected.

2.1 Summary of data received

The data were provided in three text files:

- header_Form_J_Years_1_and_2.txt: This contains responses to questions in the header of form J: DSS ID, interview time (1=AM/2=PM), symptom onset date, as well as number of people contacted in own compound for six different time points: today am, today pm, yesterday am, yesterday pm, day before yesterday am, and day before yesterday pm.
- longjtable.txt: This contains responses to contacts in other locations. There are 12 locations and 3 days, and one row for each possible location/day combination. For each location/day, we have responses to whether or not the participant visited (1=yes, 2=no), when the participant visited (1=AM/2=PM), number of people spiked with rounded to nearest increment of 5, village number, and (for compounds) compound number.
- main_trial_data_export.txt: This contains symptom and influenza test information collected from health posts and during household surveillance, as well as demographic information that was merged from the database. The information was collected from forms H (health post) and G (compound). We have whether the respondent experienced any of the following symptoms in the last 7 days: fever, cough, sore throat, nasal congestion, runny nose. We also have the date of symptom onset, whether the symptom is still present, date of clinical assessment, temperature, whether or not the cough is productive, respiratory frequency, whether the individual was referred to the doctor, and whether nasal and pharyngeal samples were collected, and malaria and type-specific influenza test results. We have the following demographic information: DSS ID, birthdate, hamlet, village, and compound ID numbers (see data cleaning notes below), birth date, gender, mother and father's ID, compound chief ID, and ethnicity.

2.2 Missing Data

A large number of participants declined to report numbers of contacts, either in all locations, or in certain locations. The code for unreported number of people contacted in a location was "99" or "999". The code "98" was used to mean "98 or more contacts". We recoded all values of 99 and 999 to NA. Histograms of numbers of people contacted at home, as well as in other locations, suggested that values of 95, 96, 97, and 98 also indicate missing values. This is because nearly no participants reported numbers of contacts between 50 and 94, but large numbers reported numbers of contacts between

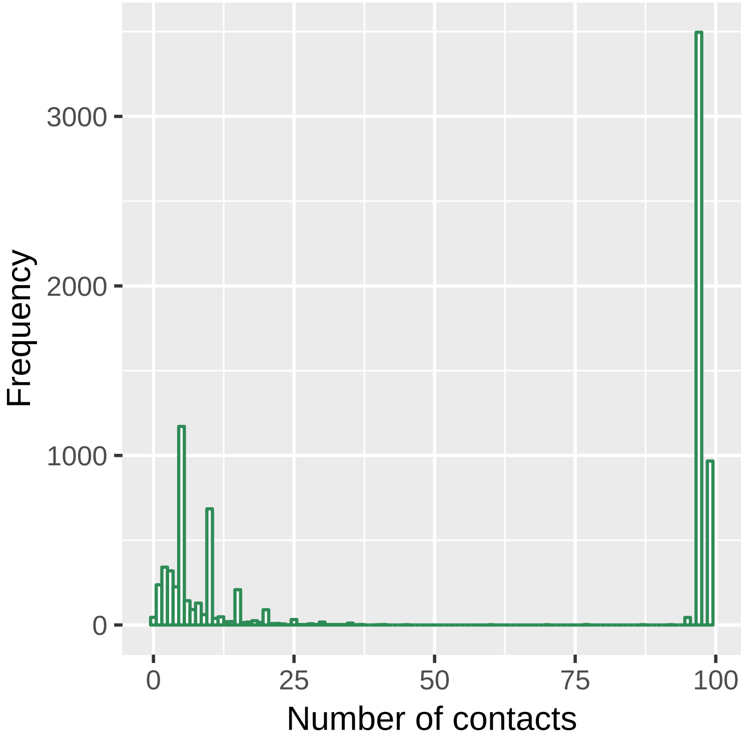

Figure I: Numbers of people contacted at home, the morning before the survey date. This figure omits reports of 99 and 999, which were missing value codes. The large numbers of reports with values between 95 and 98, and the lack of reports between 40 and 90 suggest that 95-98 were also used as missing value codes.

95 and 98. This can be seen in Fig I, which depicts number of contacts made at home the morning before the survey date. Therefore, we recoded reports between 95 and 98 to NA. Table D shows numbers of reports of 99, 999, and 95-98 in various locations, as well as numbers of non-missing reports.

2.3 Data cleaning

This section summarizes data cleaning that was done after the data was received.

2.3.1 long.j

1. The variable long.j$place contained information on the place that each participant visited outside of their compound (an integer from 1 to 12). 18 rows had a missing value for this variable, and they were removed from long.j.

2. The variable long.j$visit.time contained information on the time that each participant visited a location (1=AM, 2=PM, 3= AM and PM). There were 23 rows

Table D: Numbers of non-missing and missing reports of number of people contacted in various locations. Missing values were supposed to be coded as 99 or 999, but apparently 95–98 also indicated missing values.

	Missing value code			
Location	95-98	99	999	Non-missing
Home	3540	967	0	4060
Other compound 1	87	257	0	734
Other compound 2	21	65	0	151
Other compound 3	5	16	0	33
Other compound 4	2	8	0	8
Other compound 5	3	4	0	5
Market	83	95	0	120
Mosque/Church	6	26	0	10
Field	4	44	0	451
School	45	151	0	72
Sports field/Public place	8	89	0	82
Outside of the study zone	4	23	0	30
Another place	7	61	0	303

with a value greater than 3; these were recoded as NA.

3. The variable long.j$visit.yes.no contained information on whether a participant visited the corresponding location (1=yes and 2=no). However, there were 5 rows miscoded as a 3 but had some information on their visit while 9 rows miscoded as a 3 but had no information on their visit. The 5 rows that had some information were recoded as a 1 (did visit) while the 9 rows that had no information were recoded as a 2 (did no visit).

4. For the 71 rows that have missing values for long.j$visit.yes.no but have some information across the 4 variables that contained information on their visit, they were recoded from NA to 1.

5. To check for consistency in the data, we found that 321 rows had a 1 for long.j$visit.yes.no (yes for visited a place) but had missing info for the other 4 variables that were supposed to contain information on their visit. In contrast, 2,148 rows had a 2 for long.j$visit.yes.no (no for visited a place) but had at least 1 non-missing value for the 4 variables that contained information on their visit. In addition, it was found that about 99.3% of all rows in long.j were consistent, meaning they either had yes for visited a place and had some information on their visit, yes for visited a place but refused to provide information on their visit, or no for visited a place and had completely no information on their visit.

6. After the previous consistency check, rows that had a 2 for long.j$visit.yes.no but had some information on their visit were recoded to 1 (did visit).

7. Another consistency check was performed to determine how many rows that a long.j$spoke.number of at least 95 and long.j$visit.yes.no is 2. There were 0 rows where the condition was true, which provided evidence that the previous recoding was successful.

8. The variable long.j$spoke.number contained information on how many contacts were made at a location. There were 5,733 rows with a value of at least 95 and were recoded as NA.

9. For the 1,318 rows where long.j$visit.yes.no was 1 but long.j$visit.time was missing (visited but no information on time of visit), their long.j$spoke.number were recoded as NA due to the ambiguity of how many contacts they made in the morning and afternoon/evening.

10. For the 295,904 rows that had a value of 2 for long.j$visit.yes.no but long.j$spoke.number was missing, their long.j$spoke.number were recoded from NA to 0.

11. The variable long.j$form.number.j contained information on the form number of the J form, in which multiple rows can have the same form number as they belonged to the same person. Regularly, each form number should appear 36 times due to the 12 locations combined with the 3 days. However, there were 100 forms that did not have 36 rows. Of the 100, 2 forms had more than 36 while 98 forms had less than the 36.

12. After examining the 2 forms (7040 and 8724) that had more than 36 rows, it was found that there were duplicate rows containing the same information. Therefore, an algorithm was implemented to remove the duplicate rows.

13. A new variable called long.j$day.place was created by pasting long.j$form.day and long.j$place together. The purpose was to use this variable later when reshaping the data frame from long format to wide format. The two variables of long.j$form.day and long.j$place were then dropped.

14. It was found that there were 7 pairs of duplicate combinations of long.j$day.place and long.j$form.number.j. After examining each case, long.j$day.place was recoded correspondingly.

2.3.2 head.j

1. The variables of head.j$contacts.number.today.am, head.j$contacts.number.today.pm, head.j$contacts.number.today.am, head.j$contacts.number.yesterday.am, head.j$contacts.number.yest[illegible] head.j$contacts.number.two.days.ago.am, and head.j$contacts.number.two.days.ago.pm contained information on the number of people each participant spoke to within their own compound. It was found that the number of rows that had a value of at least 95 were 2,961, 6,056, 4,507, 4,693, 4,865, and 4,999, respectively. These were all recoded as NA.

2. The variable head.j$interview.time contained information on the time that the interview took place (1=AM and 2=PM). However, there were 82 rows with an interview time of 3 and were recoded as 2.

3. It was found that there were 68,741 blank spaces in head.j. These blank spaces were recoded as NA.

2.3.3 trial

1. The variable called trial$compound.new contained information on a new compound number for each participant but it was found to be not useful. Therefore, this variable was removed.

2. The variable called trial$fever.temperature contained information on the temperature of the fever for each participant if they had one. There were 76,900 participants with a value of 99999 and were recoded as NA.

3. The variable called trial$throat.less.than.three.days contained information on whether or not a participant had a sore throat for less than 3 days. There was 1 participant with a value of 3 and was recoded as 2.

4. The variable called trial$malaria.test.type contained information on the type of malaria test, which should only be 1 or 2. There were 44 participants with a value of 3 and were recoded as 2.

5. The variable trial$gender contained information on the gender of each participant (M=male and F=female). There were 42 participants with a value of either - or 0 and were recoded as NA. There were 6 participants with a value of f rather than F and were recoded as F.

6. The variable trial$mother.id contained information on the mother's id of each participant. There were 530 participants with a mother's id of either -1 or 0 and were recoded as NA.

7. The variable trial$father.id contained information on the father's id of each participant. There were 552 participants with a father's id of either -1 or 0 and were recoded as NA.

8. The variable trial$compound.chief.id contained information on the compound chief's id of each participant. There were 62 participants with a value of 0 and were recoded as NA.

9. There were 1,696,048 blank spaces in trial and were recoded as NA.

10. It was investigated that there were 67 pairs of rows that share the same trial$dss.id and trial$symptom.date, 1 case for 3 rows, and 1 case for 4 rows. The case for 3 rows had exactly the same information except for trial$corrected.serial.number

and 2 of the 3 rows were removed for analysis. The case for 4 rows had exactly the same information except for different trial$form.number and 3 of the 4 rows were removed for analysis. For the 67 pairs of rows, 52 pairs of rows differed only by trial$corrected.serial.number while the other 15 rows differed by other variables. For the contact network analysis, one of the two rows within each of the 67 pairs was removed.

2.4 Variable creation

Symptom status for the day before the survey was programmed as follows:

1. Asymptomatic if the reported symptom start date was the same as the survey date
2. Symptomatic if either of the following were true:
   - The symptom start date was the day before survey date or
   - The symptom start date was two or more days before survey date and at least one symptom started 'today or yesterday' or was reported as ongoing
3. Missing if the symptom start date was two or more days before survey date and no symptoms started 'today or yesterday' or were reported as ongoing

Symptom status for two days before the survey was programmed as follows:

1. Asymptomatic if the reported symptom start date was the same as the survey date or the day before the survey
2. Symptomatic if the reported symptom start date was two or more days before the survey
